# Rheumatoid arthritis patients on persistent moderate disease activity on biologics have adverse 5-year outcome compared to persistent low-remission status and represent a heterogeneous group

**DOI:** 10.1186/s13075-020-02313-w

**Published:** 2020-09-29

**Authors:** Irini Genitsaridi, Irini Flouri, Dimitris Plexousakis, Konstantinos Marias, Kyriaki Boki, Fotini Skopouli, Alexandros Drosos, George Bertsias, Dimitrios Boumpas, Prodromos Sidiropoulos

**Affiliations:** 1 Rheumatology and Clinical Immunology Department, School of Medicine at University of Crete, Voutes, 71110 Heraklion, Greece; 2 Institute of Computer Science at Foundation for Research and Technology Hellas, Heraklion, Greece; 3 Informatics Engineering Department, Technological Educational Institute of Crete, Heraklion, Greece; 4 Rheumatology Department, Sismanoglio Hospital, Athens, Greece; 5 Nutrition and Dietetics Department, Harokopio University of Athens, Athens, Greece; 6 Rheumatology Department, School of Medicine at University of Ioannina, Ioannina, Greece; 7 4th Internal Medicine Department, School of Medicine at National and Kapodistrian University of Athens, Athens, Greece

**Keywords:** Persistent moderate rheumatoid arthritis, Biologics, Functionality, Serious adverse events, Heterogeneity

## Abstract

**Background:**

The long-term outcome of rheumatoid arthritis (RA) patients who in clinical practice exhibit persistent moderate disease activity (pMDA) despite treatment with biologics has not been adequately studied. Herein, we analyzed the 5-year outcome of the pMDA group and assessed for within-group heterogeneity.

**Methods:**

We included longitudinally monitored RA patients from the Hellenic Registry of Biologic Therapies with persistent (cumulative time ≥ 50% of a 5-year period) moderate (pMDA, 3.2 < DAS28 ≤ 5.1) or remission/low (pRLDA, DAS28 ≤ 3.2) disease activity. The former was further classified into persistent lower-moderate (plMDA, DAS28 < 4.2) and higher-moderate (phMDA, DAS28 ≥ 4.2) subgroups. Five-year trajectories of functionality (HAQ) were the primary outcome in comparing pRLDA versus pMDA and assessing heterogeneity within the pMDA subgroups through multivariable mixed-effect regression. We further compared serious adverse events (SAEs) occurrence between the two groups.

**Results:**

We identified 295 patients with pMDA and 90 patients with pRLDA, the former group comprising of plMDA (*n* = 133, 45%) and phMDA (*n* = 162, 55%). pMDA was associated with worse 5-year functionality trajectory than pRLDA (+ 0.27 HAQ units, CI 95% + 0.22 to + 0.33; *p* < 0.0001), while the phMDA subgroup had worse 5-year functionality than plMDA (+ 0.26 HAQ units, CI 95% 0.18 to 0.36; *p* < 0.0001). Importantly, higher persistent disease activity was associated with more SAEs [pRLDA: 0.2 ± 0.48 vs pMDA: 0.5 ± 0.96, *p* = 0.006; plMDA: 0.32 ± 0.6 vs phMDA: 0.64 ± 1.16, *p* = 0.038]. Male gender (*p* = 0.017), lower baseline DAS28 (*p* < 0.001), HAQ improvement > 0.22 (*p* = 0.029), and lower average DAS28 during the first trimester since treatment initiation (*p* = 0.001) independently predicted grouping into pRLDA.

**Conclusions:**

In clinical practice, RA patients with pMDA while on bDMARDs have adverse long-term outcomes compared to lower disease activity status, while heterogeneity exists within the pMDA group in terms of 5-year functionality and SAEs. Targeted studies to better characterize pMDA subgroups are needed, in order to assist clinicians in tailoring treatments.

## Background

Rheumatoid arthritis (RA) disease activity state is associated with long-term prognosis [1]. According to the widely accepted treat-to-target (T2T) strategy, the aim of treatment in RA is to improve patients’ health-related quality of life by abrogation of inflammatory burden [2]. T2T approach in RA aims remission or low disease activity, evaluated by composite indexes such as the disease activity score of 28 joint counts (DAS28) [3]. Biologic disease-modifying antirheumatic drugs (bDMARDs) are potent agents which control inflammation and improve prognosis. Their clinical effectiveness has been extensively reported in controlled clinical trials and registries. Data from registries have shown that 15–35% of patients treated with TNFα inhibitors (TNFis) achieve remission at 12 months [4–7] and figures are comparable with non-TNFis such as tocilizumab [8]. Interestingly, approximately 30–50% of patients although improve disease activity, they still have moderate disease activity (MDA) after treatment with the first bDMARD [6].

Outcomes of patients with residual MDA while on treatment are available only from trials focusing on early-onset disease treated with conventional synthetic DMARDs (csDMARDs) [9, 10] and a pooled analysis of randomized controlled clinical trials of TNF is reported on early (1-year) outcomes [1]. Moreover, most of the registries report on disease activity status at prespecified time-points after treatment, while data indicative of long-term longitudinal disease activity course (i.e., time-averaged disease activity) are limited. To this end, data for RA patients on bDMARDs having persistently MDA are scant. Rheumatologists in clinical practice often confront with patients who have residual disease activity even after switching biologic disease-modifying drugs. Therefore, and given the lack of data for this group, we sought to assess the long-term outcome of RA patients on bDMARDs who exhibit persistent moderate disease activity (pMDA) in clinical practice context.

Herein, we analyzed data from the Hellenic Registry of Biologic Therapies to evaluate the long-term (5 years) outcome of RA patients on bDMARDs who exhibit pMDA. We aimed to compare the functional status (HAQ) at 5 years and its longitudinal course (trajectories) in patients with pMDA versus those on persistent lower inflammatory burden (pRLDA). Additionally, we compared serious adverse events (SAEs) occurrence during the course of the follow-up between the two groups. We finally assessed for clinical heterogeneity within pMDA and looked for early predictors of patients’ classification into the distinct persistent disease activity groups.

## Methods

### Data source

The Hellenic Registry of Biologic Therapies (HeRBT) was established in 2004 as a multicenter (7 centers) prospective observational cohort of patients with inflammatory arthritides [6]. RA and spondyloarthritis patients are included in the HeRBT at the initiation of first bDMARD and are followed-up for as long as they receive bDMARDs. Detailed analysis of protocol is given elsewhere [6, 11]. Treatment decisions (bDMARD selection, co-medication, dosage adjustments/switches) were made at the discretion of treating rheumatologists based on clinical assessments, national guidelines, and patient’s preferences. All adverse events were reported using a separate form in every visit by patients and treating physicians and details regarding their seriousness, healthcare utilization, and outcome were recorded. Ethical approval was obtained from the Institutional Review-Board of the University Hospital of Heraklion, Crete (decision number 1476/20-03-2012), along with participants’ informed consents.

### Patients

We analyzed all adult patients diagnosed with RA who were registered for the first time in HeRBT until 31 May 2013 and had continuous follow-up for at least 5 years, irrespective of bDMARD treatment switches. Data were censored when patients completed 5 years of follow-up.

### Longitudinal clinical data

Data harmonization was performed to summarize longitudinal disease activity (assessed by DAS28 index) and functionality (assessed by Health Assessment Questionnaire (HAQ)) assessments in clinically relevant therapy time (TT) intervals. Specifically, we defined a total of eight TT intervals that spanned sequentially over the 5 years follow-up period, every 6 months for the first 2 years and every year afterwards. DAS28 and HAQ averages were computed for each patient, in each TT interval, from all clinical assessments conducted in the specific time period. In addition, the patients’ serious adverse events (SAEs) were computed during the 5 years of follow-up. SAEs were defined as adverse events that required hospitalization and/or intravenous antibiotics, resulted in significant disability, and/or were life-threatening or fatal.

### Definitions and clustering by persistent disease activity

Patients were classified into persistent low (pRLDA) and persistent moderate (pMDA) disease activity groups, if they correspondingly had DAS28 ≤ 3.2 and 3.2 < DAS28 ≤ 5.1 for cumulative time percentage ≥ 50% of the 5-year follow-up time. Cumulative time percentage (CTP) of a DAS28 range was defined as the ratio of TT intervals that have DAS28 in the specific range. A patient was clustered in a persistent disease activity group when a specific DAS28-range occurred for CTP ≥ 50% or equivalently for at least 4 out of the 8 TT intervals (any of them).

To assess for within-group heterogeneity, patients with pMDA were further sub-divided into persistent lower-moderate (plMDA) and higher-moderate (phMDA) disease activity groups, when they fulfilled the additional criterion of having persistent DAS28 < 4.2 and DAS28 ≥ 4.2, respectively, for CTP ≥ 50%, as defined above. Any conflicting cases, where patients could be classified in two different persistent disease activity groups, were resolved by the preference policy of the worst-case scenario (classification in the higher disease activity group).

### Statistical analysis

Summary descriptive measures were used on baseline characteristics of the persistent disease activity groups. Non-parametric hypothesis tests (Kruskal-Wallis, Wilcoxon rank-sum, and chi-squared, as appropriate) were used to compare groups’ differences at baseline characteristics (Bonferroni-corrected *p* value = 0.0019 to account for multiple comparisons) and also to compare groups’ 5-year outcomes regarding functional status (HAQ) and cumulative serious adverse events (SAEs). To estimate the required sample size to compare functionality, we formulated the null hypothesis that functionality (HAQ) at 5 years would not differ significantly between pRLDA and pMDA groups (i.e., difference in HAQ ≤ 0.22 units). To reject this null hypothesis with 80% power (*α* = 0.05) and assuming 0.5 mean HAQ (0.5 std) in the pMDA and 0.28 in the pRLDA group and patient allocation ratio pRLDA to pMDA to be 1:2, the estimated required sample size was at least 61 and 122 patients in the pRLDA and pMDA groups, respectively.

Multivariable mixed-effect regression analysis was used to associate the persistent disease activity groups pRLDA and pMDA with different 5-year functionality trajectories. Additionally, multivariable mixed-effect regression was performed for pMDA group to assess for clinical heterogeneity (5-year functionality trajectories) between plMDA and phMDA subgroups. In the analyses, HAQ was modeled as the dependent outcome variable while the persistent disease activity group (or subgroup) was modeled as fixed-effect variable (categorical, dummy-coded with reference-category the lower persistent disease activity group). We accounted for individual patient variability with a random effect on the patient level (random intercept for each patient) and adjusted for possible confounding effects of gender, age, and disease duration (fixed-effects)*.* Time in treatment course was modeled as fixed-effect categorical variable (dummy-coded, 9-values, reference-category baseline, remaining values represent the 8 TT intervals). This time representation was selected to model the change in HAQ in each TT interval compared to baseline. Alternative representations of groups’ interactions with time did not yield significantly better results on comparison metrics of AIC, BIC, and negative log-likelihood.

Multivariable logistic regression was also performed to analyze for early predictors that classify in pMDA (versus pRLDA) group. The model was adjusted for gender, age (per year), disease duration (per year), and previous csDMARDs at baseline (binary, true for csDMARDs < 2). The model also included the following patient’s characteristics from baseline and first therapy semester: baseline disease activity (DAS28), baseline functionality (HAQ), anti-TNF treatment initiation, average disease activity in first semester (DAS28-average), and functionality difference (ΔHAQ) in first semester’s average (HAQ-average) from baseline (binary, true for difference < − 0.22). Predictive efficiency of the model was evaluated using the 10-fold cross-validation process (90% training set, 10% test set, and 10 repetitions without resubstitution) in the samples of the analysis cohort, in order to avoid overfitting and selection bias. The efficiency metrics, accuracy (ACC), sensitivity or true positive rate (TPR), specificity or true negative rate (TNR), and the area under the receiver-operating-characteristic curve (AUC), were averaged from 10 independent datasets extracted from the cross-validation process.

### Missing data management

Patients who had proportionally more missing than existing DAS28-assessments were excluded in order to maintain data quality. Missing DAS28-data in the rest of the patients were imputed with a multivariable mixed-effect regression model, due to the time-dependent nature of the longitudinal DAS28-data (repeated correlated DAS28-measurements in the same patients). The model was adjusted on gender, age, and disease duration (fixed-effects) and included a random effect on the patient level and a fixed-effect variable for time (as in the aforementioned mixed-effect model). All analyses were performed in MATLAB 9.2 statistical toolbox.

## Results

### Cohort characteristics

The analysis cohort included 385 patients from the HeRBT registry that fulfilled two criteria, (a) they had at least 5 years follow-up and (b) they exhibited persistent low or moderate disease. This cohort was selected out of the 1466 RA patients included in the registry until May 2013, after excluding 763 patients due to < 5 years follow-up, 166 patients having missing longitudinal DAS28-data > 50%, 142 exhibiting persistent high inflammatory burden, and 10 patients not exhibiting any persistent disease activity level. This was a cohort mostly with established RA (mean disease duration 9.2 years), 70 patients had disease duration < 2 years, and the mean monitoring duration was 7.5 years (Table 1). Patients were treated with an average of 2.34 (± 1.13) csDMARDs prior to first bDMARD, while 279 (72%) were on combination with methotrexate. At the end of 5 years, 208 (54%) patients received a second and 99 (26%) received a third sequential bDMARD.

**Table 1:** Baseline characteristics of patient cohort and the different persistent disease activity groups

Variable name	Cohort (*n* = 385)	Remission			
		Low pRLDA (*n* = 90)	Moderate pMDA (*n* = 295)	Lower-moderate plMDA (*n* = 133)	Higher-moderate phMDA (*n* = 162)
Females^a,b^	293 (76%)	52 (58%)	241 (82%)	102 (78%)	139 (86%)
Age (years)^a,b^	56.35 ± 13	51.96 ± 13	57.69 ± 12	55.11 ± 12.31	59.81 ± 11.59
RA duration (years)	9.2 ± 8.6	9.57 ± 10	9.1 ± 8.12	8.71 ± 7.71	9.43 ± 8.45
RA duration < 2 years	70 (18%)	22 (24%)	48 (16%)	20 (15%)	28 (17%)
Seropositive* (*n* = 217)	100 (46%)	15 (47%)	85 (46%)	37 (51%)	48 (43%)
TJC28* (*n* = 316)^a,b^	10.53 ± 6.5	7.93 ± 6.3	11.14 ± 6.4	9.61 ± 5.56	12.39 ± 6.74
SJC28* (n = 316)^a,b^	8.99 ± 6.24	6.17 ± 6.1	9.66 ± 6.1	8.14 ± 5.55	10.89 ± 6.26
ESR* mm/s (*n* = 256)	38.62 ± 24	35.37 ± 23	39.4 ± 24	37.86 ± 23.42	40.63 ± 25.02
CRP* mg/dl (*n* = 227)^a^	2.57 ± 6	4.07 ± 12	2.22 ± 3.15	2.31 ± 3.32	2.15 ± 3.02
DAS28 (imputed)^b^	5.7 ± 0.99	4.92 ± 1	5.94 ± 0.86	5.62 ± 0.74	6.02 ± 0.86
CDAI* (*n* = 296)^b^	33 ± 12.19	26.06 ± 12	34.6 ± 12	30.86 ± 10.05	37.62 ± 12.13
SDAI* (*n* = 261)^b^	35.34 ± 13	29.99 ± 17	36.64 ± 12	32.66 ± 10.44	39.63 ± 12.18
Physician VAS G.* (*n* = 298)^a,b^	69.5 ± 14.8	62.92 ± 16	71.13 ± 14	67.57 ± 15.12	73.97 ± 12.7
Patient VAS G.* (*n* = 315)^b^	66.11 ± 19	59.43 ± 25	67.69 ± 17	63.74 ± 17.82	70.93 ± 15.54
Patient VAS Pain* (*n* = 302)	67.41 ± 17	62.93 ± 25	68.5 ± 17.2	66.26 ± 18.57	70.38 ± 15.87
HAQ (imputed)^a,b^	0.84 ± 0.44	0.63 ± 0.4	0.9 ± 0.5	0.77 ± 0.43	1 ± 0.45
Euroqol* (*n* = 82)	0.34 ± 0.38	0.36 ± 0.4	0.34 ± 0.39	0.4 ± 0.34	0.26 ± 0.43
Previous csDMARDs	2.34 ± 1.13	2.11 ± 0.9	2.41 ± 1.18	2.34 ± 1.25	2.46 ± 1.11
Ongoing csDMARDs	1.15 ± 0.57	1.15 ± 0.6	1.15 ± 0.6	1.1 ± 0.63	1.19 ± 0.53
Monotherapy ^b^	32 (8%)	7 (8%)	25 (9%)	16 (12%)	9 (6%)
Methotrexate	279 (73%)	62 (69%)	217 (74%)	97 (73%)	120 (74%)
Anti-TNF ^b^	350 (90%)	88 (98%)	262 (89%)	124 (93%)	138 (85%)
Prednisolone	184 (48%)	44 (49%)	140 (48%)	71 (53%)	69 (43%)

Results presented as counts *n* (%) or mean (± sd)

*Missing data > 5%

^a^Group pRLDA is significantly different from pMDA (Wilcoxon rank-sum test *p* < 0.05)

^b^Group plMDA is significantly different from phMDA (Wilcoxon rank-sum test *p* < 0.05)

*TJC28* Tender Joint Count 28, *SJC28* Swollen Joint Count 28, *ESR* erythrocyte sedimentation rate, *CRP* C reactive protein, *DAS28* Disease Activity Score 28 ESR4, *CDAI* Clinical Disease Activity Index, *SDAI* Simplified Disease Activity Index, *VAS* visual analog scale 100, *Physician VAS G.* physician VAS global, *Patient VAS G.* patient VAS global, *HAQ* Health Assessment Questionnaire, *csDMARDs* conventional synthetic disease-modifying antirheumatic drugs, *Anti-TNF* TNFα inhibitors

### Persistent disease activity groups characteristics

**Fig. 1:**
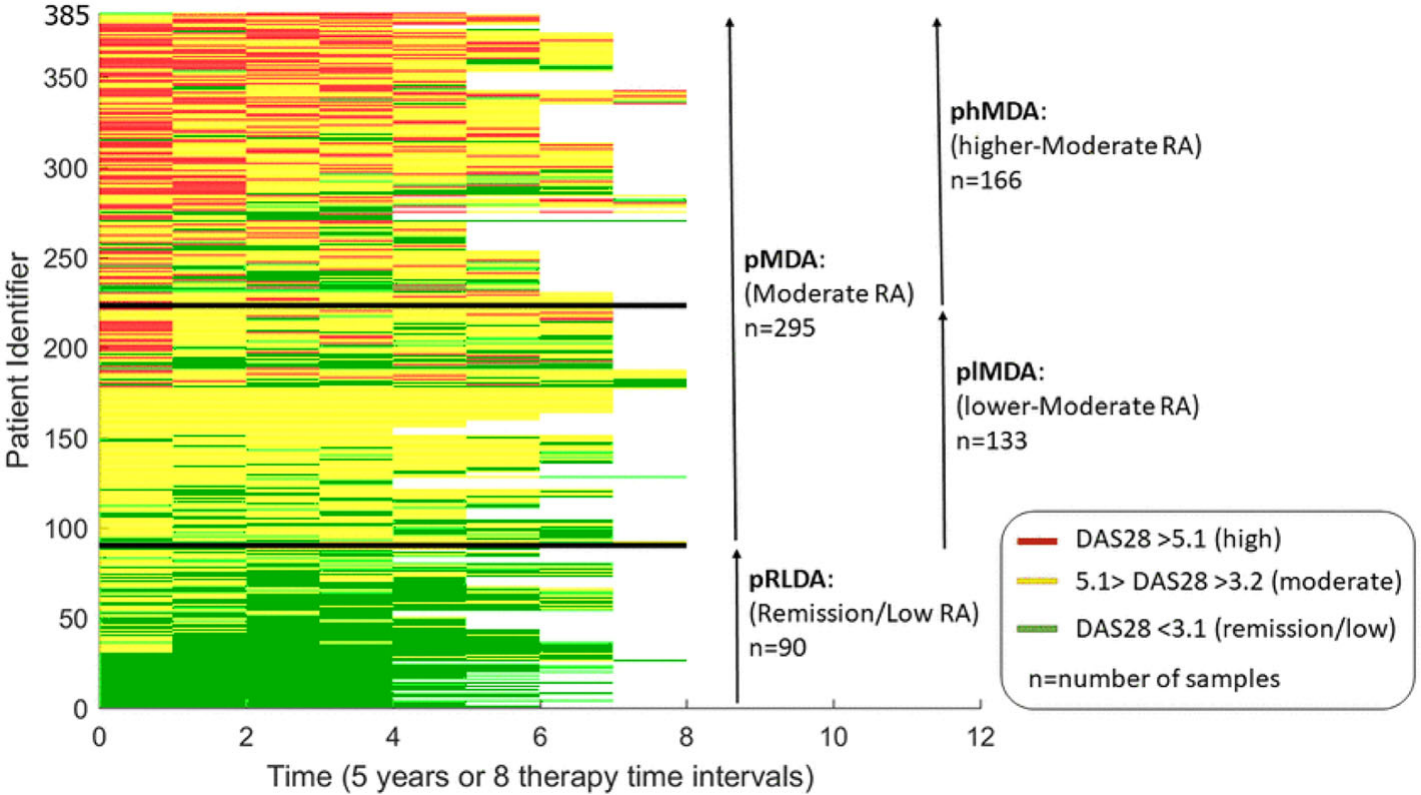
DAS28 5-year disease activity course for each cohort patient (imputed data). RA, rheumatoid arthritis; DAS28, disease activity score with 28 joint counts; pRLDA, patients with persistent remission or low disease activity; pMDA, patients with persistent moderate disease activity; plMDA, patients with persistent lower-moderate disease activity; phMDA, patients with persistent higher-moderate disease activity

**Fig. 2:**
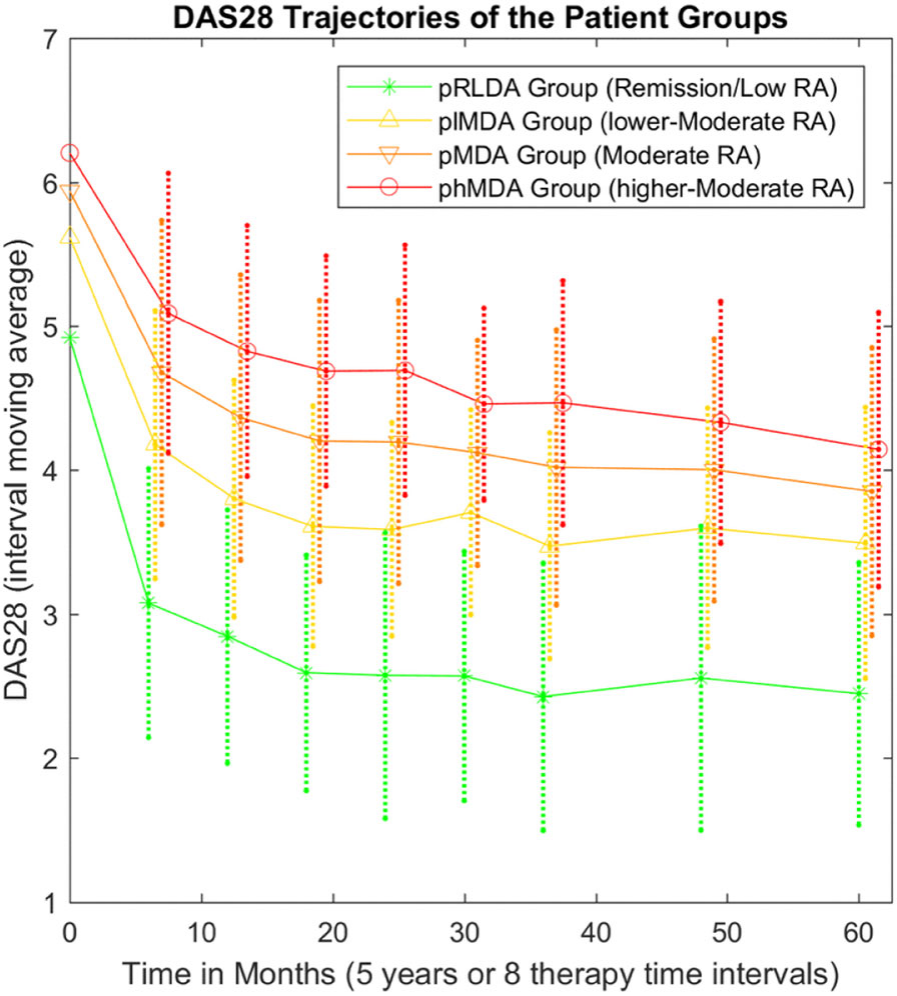
DAS28 trajectories of patient groups pRLDA (remission-low) and pMDA (moderate), and subgroups plMDA (lower-moderate) and phMDA (higher-moderate). RA, rheumatoid arthritis; DAS28, disease activity score with 28 joint counts; pRLDA, patients with persistent remission or low disease activity; pMDA, patients with persistent moderate disease activity; plMDA, patients with persistent lower-moderate disease activity; phMDA, patients with persistent higher-moderate disease activity

A total 90 (23%) and 295 (77%) patients were categorized in the pRLDA and pMDA groups, respectively. Patients in the pMDA group were further categorized in subgroups plMDA and phMDA including 133 (45%) and 162 (55%) patients, respectively **(**Table 1**)**. Patients in the pMDA group were older (58 ± 12 vs 52 ± 13 years; *p* < 0.0019 Bonferroni-corrected) and more frequently females (82% vs 58%, *p* < 0.0019) as compared to pRLDA. At inclusion, patient disease activity (mean DAS28 4.9 ± 1.0 vs 5.9 ± 0.9, *p* < 0.0019) and functionality (mean HAQ 0.63 ± 0.4 vs 0.9 ± 0.5, *p* < 0.0019) were higher in pMDA than pRLDA respectively. Analysis within the pMDA group showed that patients in the plMDA subgroup were younger (*p* = 0.0005) with lower baseline disease activity (mean DAS28 5.6 ± 0.7 vs 6.0 ± 0.9, *p* < 0.0001) than phMDA patients. A representation of the DAS28 course for each patient in the pRLDA and pMDA groups is provided in Fig. 1 while Fig. 2 presents the 5-year disease activity course (average DAS28 in the 8 TT intervals) of groups pRLDA and pMDA and subgroups plMDA and phMDA, showing clear distinct disease activity trajectories.

### pMDA group was associated with worse 5-year functionality than pRLDA

**Fig. 3:**
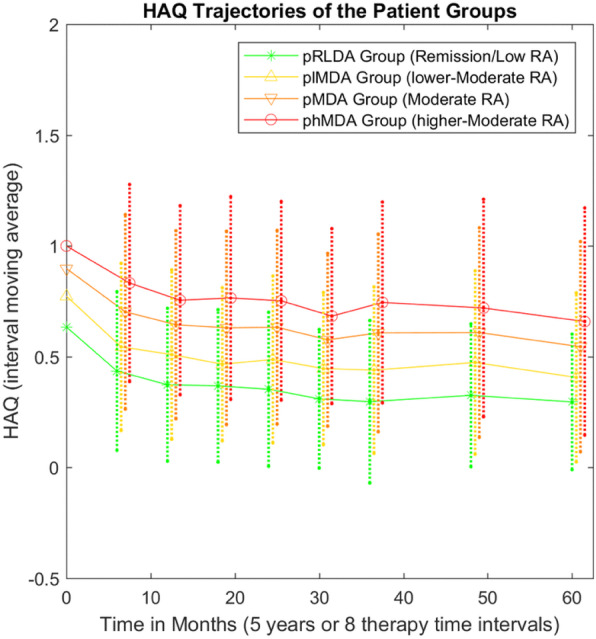
HAQ trajectories of patient groups pRLDA (remission-low) and pMDA (moderate) and subgroups plMDA (lower-moderate) and phMDA (higher-moderate). RA, rheumatoid arthritis; HAQ, functionality measured by Health Assessment Questionnaire; pRLDA, patients with persistent remission or low disease activity; pMDA, patients with persistent moderate disease activity; plMDA, patients with persistent lower-moderate disease activity; phMDA, patients with persistent higher-moderate disease activity

One of the main aims of this study was to investigate whether patients on pMDA have adverse long-term prognosis compared to patients on lower chronic inflammatory burden. The 5-year functionality (HAQ) trajectories (average DAS28 in the 8 TT intervals) of the pRLDA and pMDA groups are presented in Fig. 3, showing a clear distinct trajectory for each group. In multivariable mixed-effect regression analysis (Table 2), the pMDA group was associated with worse 5-year functionality trajectory than the pRLDA group (+ 0.28 higher HAQ trajectory in pMDA, 95% CI + 0.18 to + 0.39, *p* < 0.0001). Analysis was adjusted for possible confounding effects on gender, age and disease duration. Interestingly, the 5-year functionality was also significantly worse in females than males (+ 0.13 higher HAQ trajectory in females, 95% CI + 0.04 to + 0.23, *p* = 0.008) and in older patients (+ 0.009 higher HAQ trajectory per 1 year, 95% CI + 0.006 to + 0.012, *p* < 0.0001).

**Table 2:** Multivariable mixed-effect regression associated group pMDA with worse 5-year functionality (HAQ) trajectory than pRLDA group

HAQ multivariable analysis^†^	Coefficient^‡^	95% CI	*p* value
Group pMDA (vs pRLDA)	+ 0.28	+ 0.18 to + 0.39	*p* < 0.0001*
Gender female (vs male)	+ 0.13	+ 0.04 to + 0.23	*p* = 0.008*
Age (per year)	+ 0.009	+ 0.006 to + 0.012	*p* < 0.0001*
Disease duration (per year)	+ 0.004	− 0.001 to + 0.009	*p* = 0.092
Time 3–9 months (vs baseline)	− 0.203	− 0.279 to − 0.126	*p* < 0.0001*
Time 9–15 months (vs baseline)	− 0.289	− 0.366 to − 0.212	*p* < 0.0001*
Time 15–21 months (vs baseline)	− 0.288	− 0.364 to − 0.213	*p* < 0.0001*
Time 21–27 months (vs baseline)	− 0.285	− 0.362 to − 0.208	*p* < 0.0001*
Time 27–33 months (vs baseline)	− 0.347	− 0.432 to − 0.261	*p* < 0.0001*
Time 33–42 months (vs baseline)	− 0.326	− 0.399 to − 0.254	*p* < 0.0001*
Time 42–54 months (vs baseline)	− 0.323	− 0.396 to − 0.251	*p* < 0.0001*
Time 54–60 months (vs baseline)	− 0.375	− 0.448 to − 0.303	*p* < 0.0001*

*Variable is associated significantly with patients’ 5-year functionality (HAQ) course (*p* < 0.05 significance threshold)

^‡^Regression coefficient that represents increase (+) or decrease (−) in 5-year functionality (HAQ) course associated with the variable (category membership for categorical variable or unit increase for continuous variable)

^†^Efficiency of multivariable analysis: RMSE (Root mean square error) = 0.352, *R*^2^ (*R*-squared) = 0.573

*pRLDA* persistent remission/low disease activity group; *pMDA* persistent moderate disease activity group

At 5 years, although both groups had improved functionality as compared to baseline (HAQ decrease from baseline: − 0.375 HAQ, 95% CI − 0.448 to − 0.303, *p* < 0.0001), pMDA group had worse functionality than pRLDA (HAQ 0.3 ± 0.31 vs 0.55 ± 0.47, *p* < 0.001). Notably, most of the improvement occurred within the first 12 months of treatment (HAQ decrease from baseline in first 12 months: − 0.289, 95% CI − 0.366 to − 0.212, *p* < 0.0001), showing minimal additional improvement thereafter (12th up to 60th month).

### Subgroup phMDA was associated with worse 5-year functionality than plMDA

Clinical heterogeneity has been reported for the MDA group of patients on csDMARDs while limited data are available concerning bDMARDs. We assessed whether our cohort of RA patients on bDMARDs having persistent moderate disease activity represents a heterogeneous group. For this, we compared lower and higher pMDA subgroups, the plMDA and phMDA, respectively. The 5-year functionality trajectories (average HAQ in the 8 TT intervals) of the patients classified in plMDA and phMDA subgroups are presented in Fig. 3, revealing a clear distinct trajectory for each subgroup. In multivariable mixed-effect regression analysis (Table 3), the phMDA subgroup was associated with worse 5-year functionality trajectory than plMDA subgroup (+ 0.26 higher HAQ trajectory in pMDA, 95% CI + 0.17 to + 0.36, *p* < 0.0001). Analysis was adjusted for possible confounding effects on gender, age, and disease duration. The 5-year functionality was also significantly worse in females than males (*p* = 0.04) and in older patients (*p* < 0.0001). At 5 years, the phMDA group had worse functionality status than plMDA (HAQ 0.41 ± 0.38 vs 0.66 ± 0.52 respectively, *p* < 0.001), although both groups were associated with improved functionality as compared to baseline (HAQ decrease from baseline: − 0.38 HAQ, 95% CI − 0.463 to − 0.297, *p* < 0.0001). The differentiation of 5-year functionality between subgroups plMDA and phMDA indicates heterogeneity within the pMDA group.

**Table 3:** Multivariable mixed-effect regression associated subgroup phMDA with worse 5-year functionality (HAQ) trajectory than plMDA subgroup

HAQ multivariable analysis^†^	Coefficient^‡^	95% CI	*p* value
Group phMDA (vs plMDA)	+ 0.26	+ 0.17 to + 0.36	*p* < 0.0001*
Gender female (vs male)	+ 0.12	+ 0.01 to + 0.24	*p* = 0.04*
Age (per year)	+ 0.009	+ 0.005 to + 0.013	*p* < 0.0001*
Disease duration (per year)	+ 0.002	− 0.004 to + 0.007	*p* = 0.543
Time 3–9 months (vs baseline)	− 0.200	− 0.287 to − 0.114	*p* < 0.0001*
Time 9–15 months (vs baseline)	− 0.285	− 0.373 to − 0.196	*p* < 0.0001*
Time 15–21 months (vs baseline)	− 0.291	− 0.378 to − 0.204	*p* < 0.0001*
Time 21–27 months (vs baseline)	− 0.279	− 0.369 to − 0.190	*p* < 0.0001*
Time 27–33 months (vs baseline)	− 0.342	− 0.444 to − 0.240	*p* < 0.0001*
Time 33–42 months (vs baseline)	− 0.308	− 0.392 to − 0.225	*p* < 0.0001*
Time 42–54 months (vs baseline)	− 0.313	− 0.396 to − 0.231	*p* < 0.0001*
Time 54–60 months (vs baseline)	− 0.380	− 0.463 to − 0.297	*p* < 0.0001*

*Variable is associated significantly with patients’ 5-year functionality (HAQ) course (*p* < 0.05 significance threshold)

^‡^Regression coefficient that represents increase (+) or decrease (−) in 5-year functionality (HAQ) course associated with the variable (category membership for categorical variable or unit increase for continuous variable)

^†^Efficiency of multivariable analysis: RMSE (Root mean square error) = 0.366, *R*^2^ (*R*-squared) = 0.538

*plMDA* persistent lower-moderate disease activity group; phMDA persistent higher-moderate disease activity group

### Patient (sub)groups are associated with different serious adverse events occurrence

**Fig. 4:**
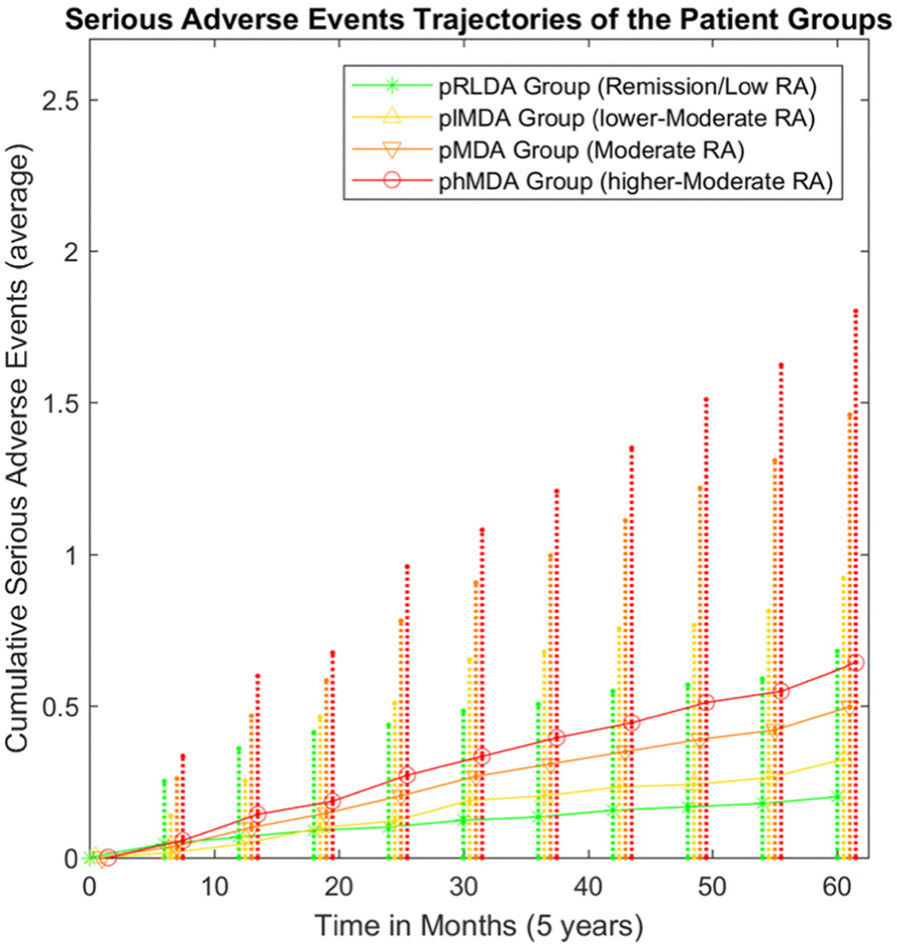
Serious adverse events trajectories of groups pRLDA (remission-low) and pMDA (moderate) and subgroups plMDA and phMDA. RA, rheumatoid arthritis; pRLDA, patients with persistent remission or low disease activity; pMDA, patients with persistent moderate disease activity; plMDA, patients with persistent lower-moderate disease activity; phMDA, patients with persistent higher-moderate disease activity

We also assessed SAEs occurrence during the course of the follow-up to estimate for any differences in the long-term outcome between distinct patients groups. The 5-year cumulative SAEs trajectories of pRLDA and pMDA groups as well as of the plMDA and phMDA subgroups are presented in Fig. 4, showing a clear distinct trajectory for each group and subgroup, respectively. At 5 years, pMDA group had higher occurrence of SAEs than pRLDA group (0.2 ± 0.48 in pRLDA vs 0.5 ± 0.96 in pMDA, *p* = 0.006). In addition, the phMDA subgroup had also higher occurrence of SAEs than the plMDA subgroup (0.32 ± 0.6 in plMDA vs 0.64 ± 1.16 in phMDA, *p* = 0.038). The differentiation between subgroups plMDA and phMDA in serious adverse events occurrence further supports the heterogeneity within the pMDA group.

### Early predictors for classification between pRLDA and pMDA groups

In view of the aforementioned clinically meaningful differences between pRLDA and pMDA patient groups, we developed a predictive model for the early classification of patients using data from patients’ early therapy months (first semester of treatment). Multivariable logistic regression analysis (Table 4) was used adjusting for possible confounding effects of gender, age, disease duration, and previous csDMARDs (at baseline). Classification in the pRLDA group (compared to pMLDA group) was associated with male gender (female gender OR 0.38, 95% CI 0.17 to 0.84, *p* = 0.017), lower baseline disease activity (DAS28 per unit: OR 0.45, 95% CI 0.29 to 0.7, *p* < 0.001), lower first semester’s average disease activity (DAS28-average per unit: OR 0.19, 95% CI 0.12 to 0.29, *p* < 0.001), and functionality improvement greater than 0.22 HAQ units in first semester’s compared to baseline (ΔHAQ < − 0.22: OR 0.38, 95% CI 0.16 to 0.91, *p* = 0.029). Performance evaluation of the model in 10-fold cross-validation process yielded 88.3% accuracy, 68% sensitivity, 95% specificity, and 91% area under the ROC curve.

**Table 4:** Multivariable logistic regression analysis to predict classification in pRLDA compared to pMDA group

pRLDA classification (vs pMDA)	Univariable analysis			Multivariable analysis^†^		
	OR	95% CI	***p*** value	OR	95% CI	***p*** value
Gender female (vs male)	0.31	0.18 to 0.52	*p* < 0.001*	0.38	0.17 to 0.84	*p* = 0.017*
Age (per year)	0.97	0.95 to 0.98	*p* < 0.001*	0.98	0.96 to 1.02	*p* = 0.26
Disease duration (per year)	1.01	0.98 to 1.03	*p* = 0.65	0.99	0.95 to 1.03	*p* = 0.68
Previous csDMARDs, count < 2	1.56	0.91 to 2.67	*p* = 0.11	1.11	0.49 to 2.5	*p* = 0.8
Anti-TNF baseline	5.54	1.3 to 23.57	*p* = 0.02*	2.9	0.51 to 16.55	*p* = 0.23
DAS28 baseline (per unit)	0.27	0.2 to 0.38	*p* < 0.001*	0.45	0.29 to 0.7	*p* < 0.001*
HAQ baseline (per unit)	0.21	0.11 to 0.4	*p* < 0.001*	0.53	0.2 to 1.38	*p* = 0.19
DAS28 1st semester (per unit)	0.17	0.11 to 0.25	*p* < 0.001*	0.19	0.12 to 0.29	*p* < 0.001*
ΔHAQ 1st semester < − 0.22	0.65	0.37 to 1.15	*p* = 0.14	0.38	0.16 to 0.91	*p* = 0.029*

*pRLDA* persistent remission/low disease activity group, *pMDA* persistent moderate disease activity group, *cDMARDs* conventional synthetic disease-modifying antirheumatic drugs, *1st semester* therapy months 3–9, *HAQ* Health Assessment Questionnaire, *ΔHAQ* HAQ difference 1st semester’s average from baseline, *ACC* accuracy, *TPR* sensitivity, *TNR* specificity, *AUC* area under receiver-operating-characteristic curve

^†^Multivariable analysis efficiency (10-fold cross-validation): ACC = 88.3%, TPR = 68%, TNR = 95%, AUC = 91%

*Below significance threshold 0.05

## Discussion

We characterized the long-term prognosis of RA patients with persistent moderate disease activity (MDA) under bDMARD treatment in the context of clinical practice. We found that a substantial proportion of patients improved disease activity status and function after treatment, yet a substantial proportion exhibited pMDA irrespective of treatment modifications. Most importantly, pMDA was associated with worse long-term (5-year) outcomes (functional limitation and serious adverse events) than persistent lower inflammatory burden. Interestingly, the subgroup with plMDA had better long-term outcomes than those with phMDA.

An important finding of this study was that persistent MDA was linked to significantly worse functionality trajectory during 5 years of bDMARDs therapy, as compared to pRLDA (Fig. 3 and Table 2). Interestingly, residual disease activity was the major contributor to HAQ increase over time (Table 2). These data are comparable to those from early RA cohorts on csDMARDs assessing the cumulative effect of disease activity on RA-related outcomes [9, 10, 12]. Although one might argue that this finding is an expected one, we considered it clinically important and novel to focus for the first time on patients treated with bDMARDs, which might exert differential immunomodulatory effects as compared to csDMARDs. For example, it has been shown that both TNFi and tocilizumab may inhibit joint destruction effectively even when residual disease activity exists, which is not the case for methotrexate [13–15].

Another key finding of our study is that MDA patients comprise a heterogeneous group in terms of outcome. Patients with plMDA have significantly better 5-year functionality trajectory than those in phMDA (Fig. 3 and Table 3). This agrees with studies focusing on early-onset RA and csDMARDs treatments, showing that patients on the lower end of MDA have significantly better outcomes than those on the higher end [10, 16]. Notwithstanding the fact that observational studies cannot provide direct support for management strategies, we consider our results to be of clinical importance and relevant to the T2T concept. Firstly, our data provide further support to the validity of T2T in clinical practice, since there was a clear superiority for all long-term outcomes in the pRLDA as compared to pMDA group. Moreover, the heterogeneity of outcomes in lower and higher MDA patients can assist T2T strategies to tailor treatments for these subgroups in order to improve outcomes.

An interesting finding was that patients on pMDA accumulated more SAEs compared to patients on pRLDA during 5 years of bDMARDs therapy (Fig. 4). Additionally, the analysis within pMDA showed that subgroup phMDA had more SAEs than plMDA at 5 years of therapy (Fig. 4). A major contributor in SAE is serious infections. The correlation between disease activity level and serious infections has been shown by several cohort studies [17–19]. Nevertheless, only our study and the analysis from the CORRONA registry by Accortt et al. are those analyzing “cumulative” disease activity levels, revealing the significant “dose effect” of inflammatory burden on the risk for serious infections [20]. Moreover, data from the Nijmegen early RA inception cohort have shown that time-averaged disease activity burden contributes to the risk of cardiovascular events in RA patients on different background therapies [21, 22]. These findings combined with the finding of higher functional decline of pMDA group underline the importance of cumulative residual disease activity as an important contributor in RA long-term prognosis.

Long-term prognosis of RA largely depends on the disease inflammatory burden and associated comorbidities. One of the limitations in the literature is that the majority of short- and even long-term studies evaluate RA-related inflammation cross-sectionally. However, values representative of longitudinal course of disease activity and its effect over time are considered to provide more valuable information. One such approach is the average disease activity from multiple years of treatment [10], while another is the area under the curve of DAS28 course (AUC) which was associated with both radiographic progression [23] and the risk for cardiovascular diseases (CVD) [21, 22]. In the present study, we applied another approach using the cumulative time percentage (CTP) that DAS28 falls within a specified range during follow-up (CTP of DAS28-range), as indicative of long-term longitudinal disease activity course. We considered the AUC method not appropriate since it may not be able to distinguish a persistent moderate disease trajectory from one fluctuating equally between low and high disease activity levels which may exhibit approximately equivalent AUC values.

Studies analyzing persistent disease activity status from cohorts of biologics have focused on persistent remission [24–30]. Herein, we focused on persistent residual disease activity (pMDA), and we found that from patients in persistent moderate or lower inflammatory burden treated on bDMARDs, 23% were classified in a persistent low or remission status while 77% still exhibited substantial inflammatory burden (pMDA) after 5 years of therapy. Even though this seems as a rather high number, yet available data from registries have shown that only 8.2–21% of bDMARDs treated patients are classified as being in persistent remission [24–27, 29]. Of note, pMDA patients in our cohort differ from pRLDA even from baseline and could be divided further in two heterogeneous subgroups.

Early predictors for patient classification in pRLDA compared to pMDA group identified in multivariable predictive modeling were male gender, lower baseline, and lower first semester disease activity (DAS28) and functionality improvement in first semester compared to baseline (ΔHAQ < − 0.22) (Table 4). Comparable to our data, a meta-analysis of six studies for factors associated to sustained remission in patients treated with TNF inhibitors showed that greater baseline disease activity, age, disease duration, baseline functional impairment, and female sex were associated with reduced likelihood of achieving sustained remission [30]. Nevertheless, prediction of response at individual level is not yet clinically available. Future studies should enrich the predictive models with additional biological parameters aiming to further increase the predictive performance of current tools.

One of the limitations of this study is patients’ missing follow-up data. In order to address missing data and also maintain data quality, we excluded patients with large percentage of missing DAS28-data (> 50%) and imputed missing DAS28-data in the rest of the patients. Of note, the imputed-dataset (385 patients) compared to the non-imputed (292 patients) included additionally 93 (24%) patients, and results were similar in both datasets (groups pRLDA and pMDA differentiated in functionality trajectory and SAEs, data not shown).

Another limitation of this study can be considered the merging of remission and low disease activity groups. The pRLDA group included 52 patients in persistent remission, 20 in persistent low disease activity, and 18 with fluctuations between low and remission disease activity. Future studies in larger datasets could focus in persistent strictly low (2.6 ≤ DAS28 ≤ 3.2) and persistent moderate (3.2 < DAS28 ≤ 5.1) disease activity comparison.

In order to evaluate the robustness of the methodological approach, we performed sensitivity analysis in shorter (3-year) therapy duration with similar results (groups pRLDA and pMDA differentiated in functionality trajectory). Furthermore, analysis on groups’ differences regarding patients’ inclusion-year (year ≤ 2007, 2007 < year ≤ 2010, year > 2010) did not yield significant variation between the groups.

### Conclusions

Our analysis revealed that a considerable proportion of RA patients on bDMARDs in clinical practice, although improve disease activity status, still manifests persistent moderate disease activity. This state was associated with adverse 5-year outcomes and was also found to present internal heterogeneity, while predictors were analyzed to assist for early patient classification. These findings further support the value of T2T strategy in order to improve long-term outcome and highlight the need for further targeted studies on persistent MDA state and its heterogeneous subgroups.

## Data Availability

The data and analytic methods that support the findings of this study are available to qualified investigators by request to the corresponding author.

## Abbreviations

RA: Rheumatoid arthritis
MDA: Moderate disease activity
pMDA: Persistent moderate disease activity
pRLDA: Persistent remission or low disease activity
plMDA: Persistent lower-moderate disease activity
phMDA: Persistent higher-moderate disease activity
HAQ: Health Assessment Questionnaire for Functionality Assessment
SAEs: Serious adverse events
bDMARDs: Biologic disease-modifying antirheumatic drugs
csDMARDs: Conventional synthetic disease-modifying antirheumatic drugs
HeRBT: Hellenic Registry of Biologic Therapies
DAS28: Disease activity score of 28 joint counts
TNFis: TNFα inhibitors
TT: Clinically relevant therapy time intervals
CTP: Cumulative time percentage
ΔHAQ: HAQ difference
ACC: Accuracy
TPR: Sensitivity or true positive rate
TNR: Specificity or true negative pate
AUC: Area under the receiver-operating-characteristic curve
T2T: Treat to target
